# Detailed causality between high‐sensitivity C‐reactive protein and recurrence of atrial fibrillation after catheter ablation

**DOI:** 10.1002/joa3.12930

**Published:** 2023-09-25

**Authors:** Naoya Kataoka, Teruhiko Imamura

**Affiliations:** ^1^ Second Department of Internal Medicine University of Toyama Toyama Japan

To Editor:

The recurrence of atrial fibrillation (AF) remains an unresolved issue. Jaroonpipatkul and colleagues evaluated the association between high‐sensitivity C‐reactive protein (hs‐CRP) and AF recurrence after catheter ablation.^1^ The authors demonstrated that higher hs‐CRP after ablation was associated with AF recurrence by performing a meta‐analysis. Several concerns have been raised.

Maintained sinus rhythm suppresses the inflammatory status. Thus, the duration and burden of AF are strongly associated with the degree of inflammatory status. One of the reasons why pre‐procedural hs‐CRP level was not associated with the AF recurrence in their study may be a variety of duration and burden of AF at baseline.^1^ It may be more appropriate to perform a pooled analysis to prepare a more homogeneous cohort, instead of performing a meta‐analysis.

It would be of great interest to find therapeutic targets associated with incremental hs‐CRP levels. An increase in hs‐CRP is reactive, and other original abnormalities should be found. For example, a variety of inflammatory‐related biomarkers have been proposed to be associated with AF recurrence, including urine isoxanthopterin,^2^ interleukin‐6, natriuretic peptide, and uric acid.^3^ Could the authors explain the reason why they focused on hs‐CRP rather than other biomarkers?

One of the major causes of AF recurrence is pulmonary vein reconnection after catheter ablation.^4^ Inflammation is unlikely to cause pulmonary vein reconnection. It may be more appropriate to exclude the patients with pulmonary vein reconnection to avoid its confounding effect.

The causality between hs‐CRP and AF recurrence is unclear. In the authors' study, postprocedural hs‐CRP level, instead of pre‐procedural hs‐CRP level, was associated with AF recurrence.^1^ The authors suggested that hs‐CRP may be an offender of AF recurrence, whereas hs‐CRP may only be a bystander of AF recurrence. What do the authors think about their causality?

## CONFLICT OF INTEREST STATEMENT

The authors declare no conflicts of interest.

## ETHICS APPROVAL STATEMENT

Not applicable.

## PATIENT CONSENT STATEMENT

Not applicable.

## CLINICAL TRIAL REGISTRATION

Not applicable.
